# The Potential Role of Ocular and Otolaryngological Mucus Proteins in Myalgic Encephalomyelitis/ Chronic Fatigue Syndrome

**DOI:** 10.21203/rs.3.rs-3171709/v1

**Published:** 2023-07-24

**Authors:** Kaylin Huitsing, Tara Tritsch, Francisco J. Carrera Arias, Fanny Collado, Kristina Aenlle, Lubov Nathason, Mary Ann Fletcher, Nancy G. Klimas, Travis Craddock

**Affiliations:** Nova Southeastern University - Fort Lauderdale/Davie Campus: Nova Southeastern University; Nova Southeastern University - Fort Lauderdale/Davie Campus: Nova Southeastern University; Nova Southeastern University - Fort Lauderdale/Davie Campus: Nova Southeastern University; Bruce W Carter Department of Veterans Affairs Medical Center: Miami VA Healthcare System; Bruce W Carter Department of Veterans Affairs Medical Center: Miami VA Healthcare System; Nova Southeastern University - Fort Lauderdale/Davie Campus: Nova Southeastern University; Nova Southeastern University - Fort Lauderdale/Davie Campus: Nova Southeastern University; Nova Southeastern University - Fort Lauderdale/Davie Campus: Nova Southeastern University; Nova Southeastern University

**Keywords:** Myalgic encephalomyelitis/chronic fatigue syndrome, mucosal-immune system, mucins, inflammation, computational modeling, systems biology

## Abstract

Myalgic encephalomyelitis/chronic fatigue syndrome (ME/CFS) is a debilitating illness associated with a constellation of other symptoms. While the most common symptom is unrelenting fatigue, many individuals also report suffering from rhinitis, dry eyes and a sore throat. Mucin proteins are responsible for contributing to the formation of mucosal membranes throughout the body. These mucosal pathways contribute to the body’s defense mechanisms involving pathogenic onset. When compromised by pathogens the epithelium releases numerous cytokines and enters a prolonged state of inflammation to eradicate any particular infection. Based on genetic analysis, and computational theory and modeling we hypothesize that mucin protein dysfunction may contribute to ME/CFS symptoms due to the inability to form adequate mucosal layers throughout the body, especially in the ocular and otolaryngological pathways leading to low grade chronic inflammation and the exacerbation of symptoms.

## Introduction/ Background

Myalgic encephalomyelitis/chronic fatigue syndrome (ME/CFS) is a serious, long-term illness characterized by a persistent, unrelenting fatigue, which is accompanied by a constellation of additional symptoms that affects many body systems. Meanwhile, the etiology of ME/CFS has yet to be fully elucidated. Among the additional symptoms tied to ME/CFS are rhinitis, sore throat, and dry eyes are quite common. Approximately, 75 to 80 percent of ME/CFS subjects appear to have an irritant rhinitis with increased mucin production [1, 2], and there appears to be a relationship between ME/CFS and dry eye syndrome [3, 4], with a previous clinical study demonstrating that sicca symptoms existed in about 70 percent of ME/CFS patients [4]. Non-exudative pharyngitis with “crimson crescents” in the posterior pharynx is also frequently seen in ME/CFS with reports that upwards of 80 percent of patients present with this symptom [5]. While other reports of related symptoms range widely (i.e. sore throat 19%-84%, cervical lymphadenopathy 23%-76%) it is clear that these symptoms are much more prevalent in ME/CFS as compared with healthy controls [6].

These symptoms may be related. A pilot study examining genome wide single nucleotide polymorphisms (SNPs) in 383 ME/CFS via the commercial company 23andMe showed ~ 70%-80% of ME/CFS subjects possess variants in genes encoding for airway, eye, and salivary mucin proteins (MUC16, MUC19, and MUC22) at 1.60 to 3.75 the reference population (see Table 1)[7]. Many of these have the potential to generate abnormal mucin proteins. For example, the Combined Annotation Dependent Depletion algorithm (CADD) [8] indicates a maximum score of 36 for MUC19 SNP rs10784618 followed by 24.7 for MUC19 rs11564109 where scores above 20 indicate that a particular SNP is predicted to be among the one percent most deleterious substitutions, indicating that this variant is highly deleterious, and a potential disease mitigating variant.

Mucin-16 and Mucin-22 are both membrane-bound mucins that are present on epithelial cells and serve as receptors and sensors to mediate signal transduction. Mucin-16, known as ovarian tumor marker CA125 due to its overexpression in ovarian and endometrial cancer [9], is present in a number of normal tissues, but mainly ocular surface epithelia such as the cornea, conjunctiva, lacrimal gland, accessory lacrimal glands, efferent tear ducts and also nose, uvula and larynx [10]. MUC16 can restrict or facilitate microbial invasion at the apical surface of the epithelium [11]. For example, it has been shown that there is greater binding of *Staphlylococcus aureus* to *in vitro*-cultured corneal cells when MUC16 is depleted [12]. Mucin-22 is a relatively novel membrane-bound mucin with previously unknown pathophysiological roles [13, 14]. Recent work indicates that mucin-22 appears to play an important protective role against severe coronavirus disease (COVID)-19 infection, with certain variants offering improved protection [15]. These variants in MUC22 however did not include those observed in Table 1. Another variant in MUC22 not listed in Table 1 appears to be associated with the risk of childhood asthma [16]. While the role of ME/CFS associated variants in MUC22 are unknown, evidence suggests they may affect the respiratory tract and response to environmental pollutants or pathogens. The variants in Table 1 for both MUC16 and MUC22 result in amino acid changes in the extracellular region of the membrane bound protein. The highly glycosylated extracellular mucin domains form a tight mesh structure that protects cells by bind pathogens to inhibit invasion [17]. It is known that serine and threonine repeats in this region are the sites of glycosylation [18], thus if these variants produce functional changes in these mucin proteins, it would therefore be in the ability of these mucins to interact with and bind pathogens.

Mucin-19 is a secreted gel forming mucin that has been detected in the submandibular gland, sublingual gland, respiratory tract, eye, and middle ear epithelium. The MUC19 SNP rs10784618 is a nucleotide change of cytosine to adenine in chromosome 12 at position 40441153. This change is a coding region of the gene resulting in a nonsense point mutation causing a premature stop codon at cysteine residue 1238 in the mucin-19 amino acid protein sequence. As the mucin-19 protein is typically 8,384 amino acids long, this mutation results in a severely truncated, incomplete, and nonfunctional protein product that is incapable of forming a proper protective barrier. The MUC19 SNP rs11564109 variant is a missense coding sequence variant resulting in an amino acid change at position 1411 from cysteine to tyrosine. While the significance of this alteration is uncertain, it may affect overall protein conformation as cysteine plays an important role in protein structure through the formation of disulfide bonds. The remaining variants likewise result in amino acid changes, although the consequence of the substitutions is not clear as currently the three-dimensional structure of MUC19 is not known.

## The Hypothesis

**Figure 1:**
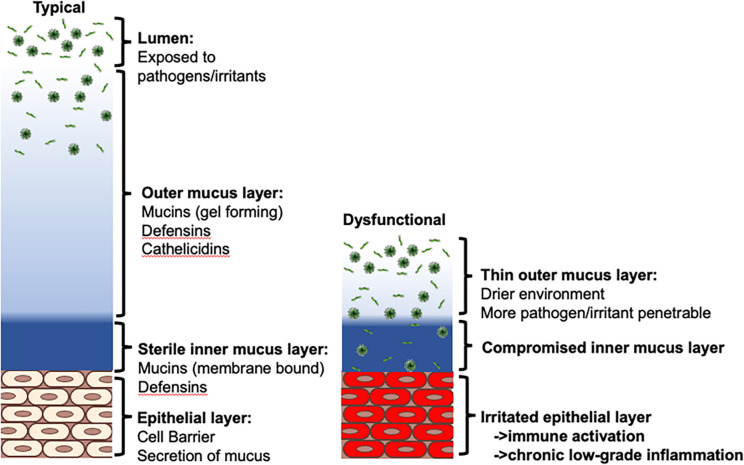
The mucosal protective barrier. (Left) Pathogens present in the lumen are prevented from reaching the epithelium via a first layer of gel-like mucins and antibacterial peptides, and a second layer of membrane bound mucins. (Right) Dysfunctional outer and inner mucin proteins lead to a compromised inner mucus layer and irritated epithelium.

The role of these mucus proteins (MUC16, MUC19 and MUC22) in the ear, nose, throat, respiratory tract, and eye is to protect and prevent infection. Exposure to the environment risks exposure to viral and bacterial pathogens (Fig. 1). The first layer of protection from these pathogens is the outer mucus layer formed of gel like mucins (such as mucin-19), and anti-bacterial peptides such as defensins and cathelicidins. This outer mucus layer serves to protect the second inner mucus layer adhered to the epithelium, keeping it sterile to avoid irritating the epithelial layer (Fig. 1 – left). Dysfunctional changes that decrease the outer gel layer of mucus (i.e., through non-functional mucin-19), coupled with an inner mucus layer incapable of binding pathogens (i.e., dysfunctional mucin-16 and mucin-22) would result in a compromised mucus layer leading to a chronic irritated epithelial layer (Fig. 1 – right).

**Figure 2:**
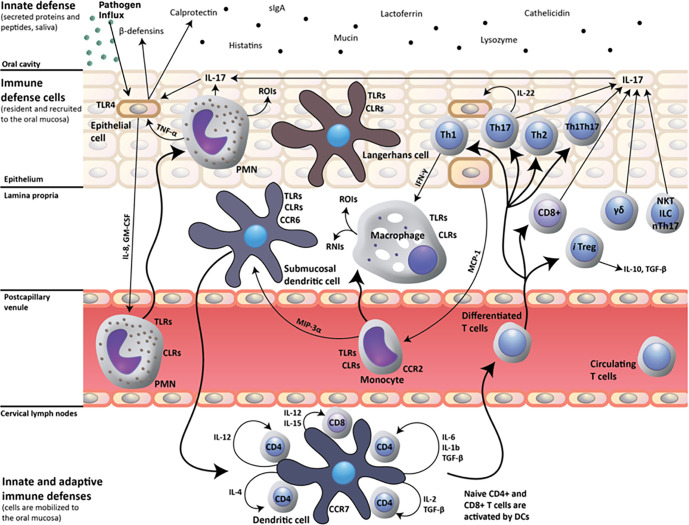
Host response to oral pathogen influx. A protective host response to infection is dependent on dendritic cell-mediated induction of Th17 cell-mediated adaptive immunity, which, by the production of interleukin (IL)-17 upregulates the innate expression of mucosal antimicrobial peptides (β-defensins, calprotectin) by epithelial cells. IL-17 also up-regulates IL-8 and granulocyte-macrophage colony-stimulating factor (GM-CSF) production by epithelial cells, which in turn trigger recruitment of polymorphonuclear neutrophils (PMN) to the oral mucosa. Innate-like cell populations, including γδ T-cells, Natural Killer T cells (NKT), innate lymphoid cells (ILC) and natural Th17 cells (nTh17), also produce IL-17 and may participate in the mucosal host response. CLRs, C-type lectin receptors; RNIs, reactive nitrogen intermediates; ROIs, reactive oxygen intermediates; and TLRs, toll-like receptors. Image adapted from [19] under the Creative Commons Attributions License 4.0 International (CC BY 4.0).

When pathogens manage to cross the mucus layer the response by the immune system from resident and recruited immune defense cells containing T-cells, dendritic cells, and macrophages, and further from the immune system within the blood comprising of peripheral blood mononuclear cells (Fig. 2) would result in a chronic low-grade inflammation. Expanding on the roles of the mucosa and cytokines within the ocular and otolaryngological environment, there is speculation as to the resulting deficits in the MUC16, MUC19 and MUC22 proteins. While preliminary studies have shown that numerous individuals have variants in these proteins, it is hypothesized that these variants cause dysfunction in the mucosal protective barrier. A dysfunctional mucosal barrier will result in a compromised barrier between epithelial cells and the environment. Smaller or weaker barriers permit pathogen access and infiltration leading to a persistent low-grade inflammation in which cytokines will be consistently released contributing to continuous sickness behaviors like those seen with ME/CFS.

## Evaluation of the Hypothesis

### Discrete ternary logic analysis of regulatory network:

**Figure 3:**
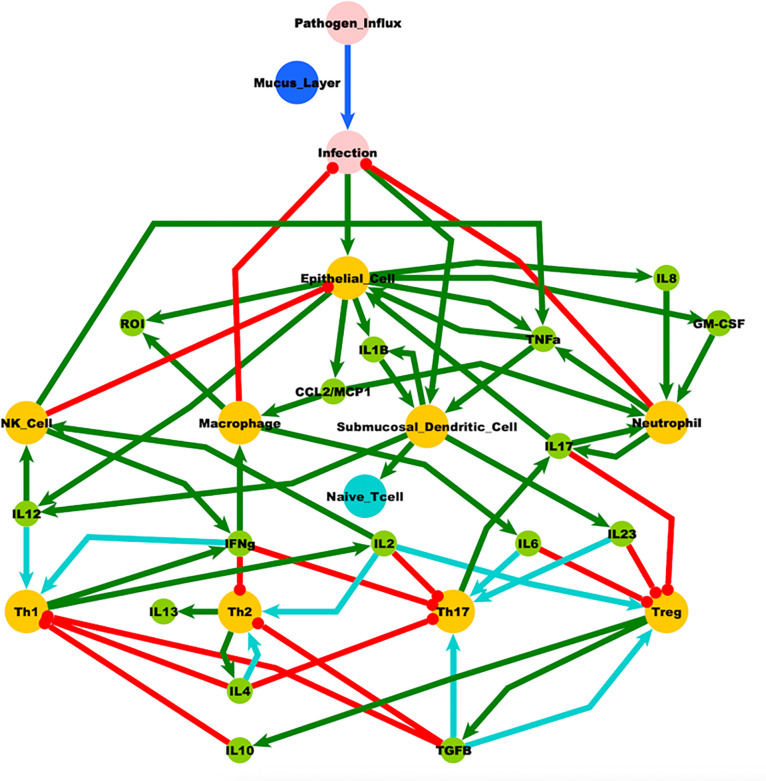
Mucosal-immune system. Nodes: Yellow, distinct immune cell types; Green, immune signaling molecules; Red, environmental pathogens. Edges: Red, inhibition; Green, stimulation. Conditionals: Cyan, Naïve T Cell dependent stimulation; Blue, Mucus layer dependent stimulation. IL – interleukin; GM-CSF - granulocyte-macrophage colony-stimulating factor; ROI – reactive oxygen intermediary; NK – natural killer cells; CCL2/MCP1 - chemokine ligand 2/monocyte chemoattractant protein 1; TNFa – tumor necrosis factor a; TGFB – transforming growth factor b; Th – T helper cell; Treg – T regulator cell.

Our previous work [20–23] suggests that the complexity of the mucosal-immune signaling system can allow for multiple regulatory modes beyond what is typically considered typical health. To provide a theoretical framework for our hypothesis here we compile molecular and cellular signaling information from various studies and reviews in the literature to create a logically consistent, theoretical model of a general ocular and otolaryngological mucosal-innate immune signaling system to explore the role of mucus protection in the homeostatic regulation of the innate immune system and the perpetuation of chronic low-grade inflammation (see Fig. 3). Logic rules are applied to this connectivity diagram to predict the system’s homeostatic behavior. Using a similar approach reported in [21, 22, 24–26] the mucosal-immune system in Fig. 3 was captured as a logic connectivity model consisting of interconnected nodes with three discrete states: −1 (suppressed), 0 (normal) and + 1 (increased). In brief, the state of the system at a point in time was described by an assignment of discrete states to all nodes. The state that each node in the system transitions to in the next time step was determined from a set of balanced ternary logic statements (see [21]), the node’s current state, and the defined interactions (i.e. activate or inhibit) of the neighboring input nodes. The logic is such that an increase in activators raises the node value, while a decrease in inhibitors decreases the value. In cases where both activators and inhibitors were increased, the node value remained unchanged. While the number of activators and/or inhibitors for a given variable may remain static, they may also be allowed to change based on predefined conditions, such as the state of one or more variables as described in [27]. In the system described in Fig. 3 conditional edges are dependent on the state of Naïve T Cells. The system is updated asynchronously (allowing only one variable to change at a time), such that for each current state there are potentially several subsequent states towards which it may evolve. The number of states, and the values they can be assigned, determine the total number of states available to the model system. By analyzing all possible states of the system, a temporal sequence of states was discerned. Steady states were defined as those states for which the current system state did not evolve in time. The steady states of the mucosal-immune system are given in Table 2. Beyond normal homeostatic regulation, our model predicts alternate self-perpetuating conditions consistent with chronic inflammation. Three stable states are shown in Table 1 with SS0 corresponding to a typical healthy state, while both SS1 and SS2 present with a stable altered Th1 immune profile. As such, these simulations of pathogen influx with deficient mucus protection were shown to be theoretically capable of forcing the system to a state of immune activation supporting a potential role for the mucosal-immune signaling system’s own homeostatic drive in perpetuating chronic low-grade inflammation.

### Comparison to ME/CFS Cytokine Panels:

To determine the applicability of this model to ME/CFS in specific, we compared our model predicted homeostatic stable states to cytokine signaling profiles in blood of female subjects with ME/CFS. Clinical data obtained as part of a larger on-going study investigating changes in cytokines in ME/CFS was used as a basis for comparison with the predicted resting states (see [28]). A total of 65 female subjects (29 with ME/CFS, 36 healthy controls), and 53 male subjects (25 with ME/CFS, 28 healthy controls) were selected without exclusion for ethnicity from the patient population within the Institute for Neuroimmune Medicine at Nova Southeastern University (NSU) in Fort Lauderdale, Florida, directed by Nancy Klimas, M.D. All subjects signed an informed consent approved by the Institutional Review Board (IRB) of NSU, Fort Lauderdale, Florida. Included subjects presented with acute onset and with an illness duration of at least four years. ME/CFS was diagnosed according to current research case definitions [29, 30]: fatigue of greater than six months duration and at least four of eight symptoms including exercise-induced relapse, myalgia, arthralgia, headache of a new and different type, nonrestorative sleep, cognitive complaints, sore throat, and tender lymph nodes. All ME/CFS study subjects presented with a 36-short form health survey (SF-36) summary physical composite score below the 50th percentile, based on population norms.

**Figure 4:**
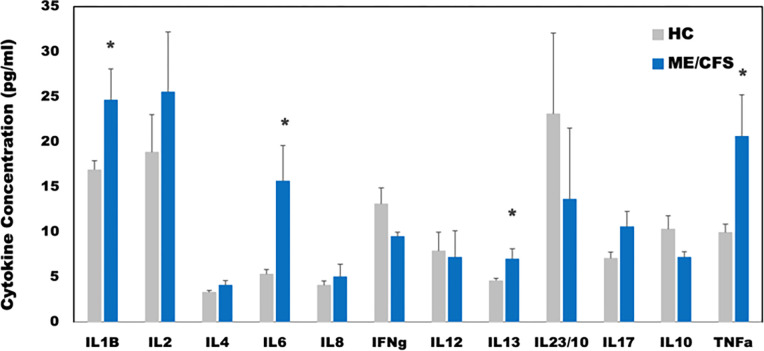
Plasma cytokine concentrations at rest for female ME/CFS subjects compared to healthy sedentary controls. * indicates p < 0.05 for two-tailed heteroscedastic t-test. Note: IL23 concentration is reduced by a factor of 10 to fit on the graph scale.

**Figure 5:**
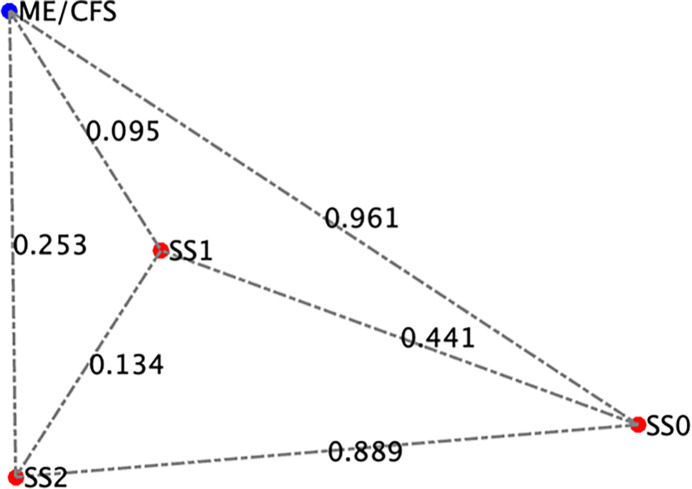
Projections of the comparison between the model predicted stable states and female ME/CFS cytokine profiles.

**Figure 6:**
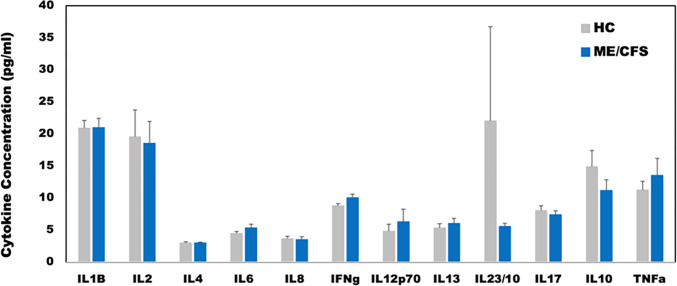
Plasma cytokine concentrations at rest for male ME/CFS subjects compared to healthy sedentary controls. * indicates p < 0.05 for two-tailed heteroscedastic t-test, no significant differences found. Note: IL23 concentration is reduced by a factor of 10 to fit on the graph scale.

**Figure 7:**
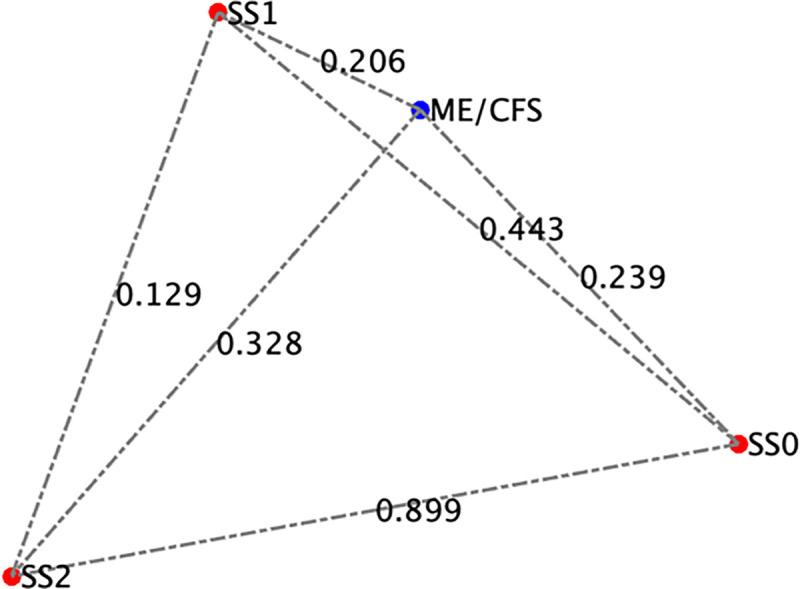
Projections of the comparison between the model predicted stable states and male ME/CFS cytokine profiles.

Healthy controls were self-defined as sedentary (no regular exercise program, sedentary employment). Plasma concentrations of IL-1β, IL-2, IL-4, IL-6, IL-8, IL-10, IL-12p70, IL-13, IL-17, IL-23, IFNγ and TNFα were measured via Q-Plex multiplex ELISA (Quansys Biosciences, Logan, Utah) from blood obtained at rest (see Fig. 5). A meta-analysis was used to calculate the significance of similarity between the inflammatory profiles of subjects with ME/CFS, and the equilibrium states predicted by the logic model. To do this the cytokine profiles were compared to each model-predicted steady-state behavior of the mucosal-immune system through the application of Brown’s theoretical approximation [31] of Fisher’s statistics, as conducted in our previous work [20–23, 27, 32]. This method was chosen as it provides a meta-analysis technique to combine non-independent probabilities and obtain an overall significance measure P based on a set of *p*-values obtained from independent *t*-tests. The aggregate value P ranges between 0 and 1, with 0 indicating complete overlap and 1 being the farthest distance from a stable state. This method is applicable as the model elements do not express independently, as evidenced by the connectivity of the mucosal-immune interaction model (Fig. 3). The above-mentioned cytokine data were compared against the model predicted states based on the 12 measured variables. To visualize the comparison of the measured states with the model-predicted stable states the multi-dimensional co-expression profiles (Figs. 4 and 6) were projected into a two-dimensional space using multidimensional scaling as done previously [27] (see Figs. 5 and 7). Here, the dissimilarity matrix defined by the aggregate P value is scaled such that the 2D Euclidean distances between points approximate the corresponding dissimilarities. This is performed using the function *mdscale* in MATLAB to minimize Kruskal’s stress criterion normalized by the sum of squares of the dissimilarities. After comparing the stable states in Table 1 with the female cytokine profiles of ME/CFS patients (Fig. 4), we found that SS1 was the most closely aligned with the ME/CFS profile (Fig. 5). The SS1 state is characterized by increased pro-inflammatory cytokines, activation of the innate immune cells and a shift towards Th1 immunity. This is consistent with our hypothesis that ME/CFS presents with a low-grade inflammatory profile. However, when comparing the male cytokine profile of ME/CFS subjects (Fig. 6), while difference was observed from healthy control, it was found to align near equidistant from health (SS0) and the SS1 state, with the SS1 being slightly more favorable (Fig. 7). This is consistent with previous work suggesting a sex difference in males with ME/CFS [33–38].

## Consequences of the Hypothesis and Discussion

The ocular and otolaryngological mucus layers normally act to protect the epithelial tissue from irritants, microorganisms and pathogens entering the body. Changes in the mucus layer lining can often be symptoms of illness such as diabetes, human immunodeficiency virus (HIV), vitamin deficiency or even neurodegenerative illnesses such as Alzheimer’s and Parkinson’s diseases. Here we have presented a hypothesis that the symptoms observed in the chronic illness of ME/CFS may, in part, be associated with a compromised ocular and otolaryngological mucus layer leading to increased likelihood of irritation of the epithelial layer in these regions resulting in a constant low-grade inflammation. This is consistent with findings indicating a preponderance of rhinosinusitis symptoms in subjects with unexplained chronic fatigue and bodily pain [39].

Chemical sensitivities are recognized as a common symptom of ME/CFS [30, 40] with multiple chemical sensitivity being a common comorbidity in the illness [30, 41]. Triggers include pesticides, perfume and petrochemicals, and natural irritants like mold and wood-fire smoke, and can lead to symptoms of headache, migraine, cognitive impairment, dizziness, fatigue, nausea, vomiting, cardiac abnormalities, skin rashes, asthma, and anaphylaxis [42] all of which are common symptoms of ME/CFS. A dysfunctional ocular and otolaryngological mucus layers would lead to a sensitive epithelium that may be irritated due to environmental exposures (i.e. chemical or biological) leading to “flares” of symptoms as the immune system is further triggered. This is consistent with the “kindling” theory of ME/CFS [43].

ME/CFS has also been associated with exposure to infectious agents, and there have been multiple reported “outbreaks” of illness [44]. Various bacteria, including members of the gut microbiome, and viruses such as human parvovirus B19, enteroviruses, as well as the herpesviruses Epstein-Barr virus (EBV), human herpesvirus-6 types A and B (HHV-6), and human cytomegalovirus (HCMV), have been implicated as possible etiological pathogens of ME/CFS [45, 46]. The symptom similarities between Long COVID (post-acute sequele of SARS-COV-2 infection) and ME/CFS also suggest that COVID-19 may play a similar role in disease onset [47]. These pathogens are all found in, and can be transmitted, by saliva or respiratory droplets. A compromised otolaryngological mucus layer would allow for increased risk of initial infection. Furthermore, the herpes viruses (i.e. EBV, HHV-6, and HCMV) remain latent within the body within salivary glands and epithelial cells and occasionally are reactivated [48]. Potential triggers of this reactivation include environmental irritants leading to inflammation [49, 50]. As such, increased irritation in these regions due to a dysfunctional mucus protective barrier would lead to a greater incidence of viral reactivation and its associated symptoms.

Many studies of ME/CFS have found evidence of reduced natural killer (NK) cell function [51–56]. In addition, some studies indicate NK cell function correlates with illness severity [57, 58]. The reason for this reduced function is unknown. NK cell activation is triggered by inflammatory mediators, cytokines, and chemokines, including IL-2 and IL-12 following recognition of stressed, and infected cells which leads NK cells to lyse target cells and secrete IFN-γ and TNF-α [59]. The reduced ability of NK cells to clear infected and stressed epithelial cells coupled with the proposed increased propensity of the epithelium to be irritated and infected in ME/CFS due to dysfunction in the mucus barrier is expected further exacerbate the problem leading to an increase in associated symptoms with a decrease in NK cell function consistent with literature.

Similar to our findings presented here, many studies indicate a sex difference in ME/CFS [33–38] with the female sex at greater risk for developing the illness and suffering endocrine events over the illness course [38]. Females with ME/CFS report greater irregularities in their menstrual cycles, with menopause and pregnancy affecting their symptomatology [38]. While the mucins discussed cluster in the ocular and otolaryngological areas, mucin-16 also is present in the endometrium with its overexpression in ovarian and endometrial cancer denoting it as a known ovarian tumor marker [9]. It has also been shown that dexamethasone, as a synthetic steroid similar to the stress hormone cortisol, upregulates the expression of mucin-16, suggesting a link between its expression and stress [60, 61]. Should the variants in mucin-16 noted here result in a dysfunctional mucus barrier it may explain these symptoms in females with ME/CFS and may contribute to the overall sex differences observed.

Overall, we have presented genetic evidence suggesting a dysfunctional mucus barrier in most individuals with ME/CFS. This dysfunction, in conjunction with environmental exposures to chemical and biological triggers, potential latent viral infection, and decreased NK cell function are expected to the overall triggering of symptoms in ME/CFS. Future work investigating the role of the ocular and otolaryngological mucus layer are ultimately needed to confirm this hypothesis.

## Figures and Tables

**Table 1:** Single Point Mutations in ME/CFS Mucin Proteins Compared to 1000 Genome Reference Population

Gene Name	rsID	ME/CFS %	1000 Genome %	Ratio	Effect	Scaled CADD Score	Top % of Deleterious Changes	Mucin Type	Location
MUC16	rs7245949	0.57	0.23	2.53	missense T >I	12.17	6.09	Membrane Bound	Airways, Eye, Reproductive Organs, Mesothelium
MUC16	rs1862462	0.50	0.25	2.02	missense S > L	3.051	49.57		
MUC16	rs2547072	0.50	0.25	2.02	missense T >I	0.635	86.41		
MUC16	rs1867691	0.50	0.22	2.30	missense I >V	0.009	99.79		
MUC19	rs10784618	0.77	0.48	1.60	stop_gained	36	0.03	Gel Forming	Airways, Eye, Middle Ear, Salivary Glands
MUC19	rs11564109	0.24	0.08	2.85	missense C >Y	24.7	0.34		
MUC19	rs2588401	0.80	0.48	1.67	missense A >T	7.453	18.01		
MUC19	rs10878538	0.26	0.11	2.33	5' UTR	4.114	38.82		
MUC19	rs1019709	0.82	0.40	2.05	intron	1.241	75.17		
MUC19	rs2588402	0.80	0.48	1.67	missense A >T	0.069	98.43		
MUC22	rs10947121	0.60	0.45	1.33	missense L >P	5.542	27.95	Membrane Bound	Airways, Eye, Middle Ear, Salivary Glands
MUC22	rs3094672	0.75	0.20	3.67	missense S >T	0.659	85.94		
MUC22	rs9262549	0.72	0.19	3.75	missense S >T	0.27	93.98		

**Table 2:** Stable Behaviors of the Mucosal-Immune System

Nodes	SS0	SS1	SS2
**Submucosal Dendritic Cell**	0	1	1
**Infection**	0	0	0
**Epithelial Cell**	0	0	−1
**IL1b**	0	1	1
**TNFa**	0	1	0
**IL12**	0	1	1
**IL8**	0	0	−1
**CCL2/MCP1**	0	0	−1
**GM-CSF**	0	0	−1
**Naive Tcell**	0	1	1
**Th1**	0	1	1
**Th2**	0	0	−1
**Th17**	0	0	−1
**Treg**	0	0	−1
**IFNg**	0	1	1
**IL2**	0	1	1
**IL6**	0	1	1
**TGFb**	0	0	−1
**IL4**	0	0	−1
**IL10**	0	0	−1
**IL23**	0	1	1
**IL17**	0	1	0
**IL13**	0	0	−1
**Neutrophil**	0	1	0
**ROI**	0	1	1
**Macrophage**	0	1	1
**NK Cell**	0	1	1

## Data Availability

The datasets used and/or analyzed during the current study available from the corresponding author on reasonable request.

## Abbreviations

CADD: combined annotation dependent depletion algorithm
CCL2/MCP1: chemokine ligand 2/monocyte chemoattractant protein 1
CLRs: C-type lectin receptors
COVID: corona virus disease
EBV: Epstein-Barr virus
GM-CSF: granulocyte-macrophage colony-stimulating factor
HCMV: human cytomegalovirus
HHV6: human herpesvirus-6 types A and B
IL: interleukin
ILC: innate lymphoid cells
ME/CFS: myalgic encephalomyelitis/chronic fatigue syndrome
NK: natural killer
NKT: natural killer T
NSU: Nova Southeastern University
nTh17: natural Th17 cells
PMN: polymorphonuclear neutrophils
RNIs: reactive nitrogen intermediates
ROIs: reactive oxygen intermediates
SF-36: 36-item short form health survey
SNP: single nucleotide polymorphisms
SS: steady state
TGFB: transforming growth factor b
Th: T helper cell
TLRs: toll-like receptors
TNFa: tumor necrosis factor a
Treg: T regulator cell
